# Supplementary material for: Radiotherapy Plan Quality Assurance in NRG Oncology Trials for Brain and Head/Neck Cancers: An AI-Enhanced Knowledge-Based Approach
Source: Cancers (Basel). 2024 May 25;16(11):2007. doi: 10.3390/cancers16112007 (PMC11171017; doi:10.3390/cancers16112007)
Supplement: Supplementary file 1 [file cancers-16-02007-s001.zip › cancers-2973047-supplementary.pdf]

Supplementary Material

Supplementary Table S1. NRG Oncology BN001 structure dosimetric compliance criteria

Structures	Parameter	Per Protocol	Variation acceptable
PTV_5000	D95%[Gy(RBE)]	=50	47.5
PTV_7500	D95% [Gy(RBE)]	<=75.75	78.75
	D95% [Gy(RBE)]	>=74.25	71.25
	D90% [Gy(RBE)]	<=78.7	81.4
	Dmax [Gy(RBE)]	<=80	82.5
SpinalCord	Dmax [Gy(RBE)]	<=50	50
BrainStemCore	Dmax [Gy(RBE)]	<=55	60
BrainStemSurf	Dmax [Gy(RBE)]	<=55	64
OptChiasm_PRV	Dmax [Gy(RBE)]	<=55	60
OptNrv_R/L_PRV	Dmax [Gy(RBE)]	<=55	60
Retina_R/L	Dmax [Gy(RBE)]	<=45	50
Brain	D5% [Gy(RBE)]	<=78.7	81.4
Lens_R/L	Dmax [Gy(RBE)]	<=7	10

Dmax is defined for a volume less than or equal to 0.03cc.

Supplementary Table S2. NRG Oncology HN001 structure dosimetric compliance criteria

Structures	Parameter	Per Protocol	Variation acceptable
PTV_High	V100%[%]	=95	>=90
	D99%[%]	>=93	>=90
	D0.03cc[%]	<=115	<=120
PTV_Intermediate1	V63Gy[%]/V62.7Gy[%]	>=95	>=90
PTV_Intermediate2	V59Gy[%]/V59.4Gy[%]	>=95	>=90
PTV_Low	V56Gy[%]	>=95	>=90
BrainStem	D0.03cc[Gy]	<=54	<=60
SpinalCord	D0.03cc[Gy]	<=45	<=50
OptNrv_R/L	D0.03cc[Gy]	<=54	<=60
OptChiasm	D0.03cc[Gy]	<=54	<=56
Mandible	D0.03cc[Gy]	<=70	<=75
TMjoint_R/L	D0.03cc[Gy]	<=70	<=75
BrachialPlexus_R/L	D0.03cc[Gy]	<=66	<=70
TemporalLobe_R/L	D0.03cc[Gy]	<=70	<=72
Parotid_R/L	Mean [Gy]	<=26	<=33

PTV_High: PTV_6996 or PTV_6996_Eval for 33 fractions; PTV_7000 or PTV_7000_Eval for 35 fractions.

PTV_Intermediate1: PTV_6270 or PTV_6270_Eval for 33 fractions; PTV_6300 or PTV_6300_Eval for 35 fractions.

PTV_Intermediate2: PTV_5940 or PTV_5940_Eval for 33 fractions; PTV_5900 or PTV_5900_Eval for 35 fractions.

PTV_Low: PTV_5600 or PTV_5600_Eval.

Supplementary Table S3. NRG BN001 Rapid Plan Model Parameters

Structures	Objectives	RP Photon Model			RP Proton Model		
		Volume(%)	Dose(Gy)	Priorities	Volume(%)	Dose(Gy)	Priorities
PTV_5000	Lower	95	51	120	97	51	200
PTV_7500	Upper	0	77	150	0	79	200
	Lower	10	77	100	99	77	200
PRV	Upper	97	75	120	0	70	80
PTV_5000 opt	Upper	0	75	100	5	65	80
Ring	Upper	n/a	n/a	n/a	0	70	100
Spinal Cord	Upper	0	45	150	0	50	100
	Line	Model Generate	Model Generate	Model Generate	Model Generate	Model Generate	Model Generate
Brainstem Core	Upper	0	50	130	0	50	130
	Line	Model Generate	Model Generate	Model Generate	Model Generate	Model Generate	Model Generate
Brainstem Surf	Upper	0	50	130	0	50	130
	Line	Model Generate	Model Generate	Model Generate	Model Generate	Model Generate	Model Generate
OptChiasm_PRV	Upper	0	50	120	0	50	120
	Line	Model Generate	Model Generate	Model Generate	Model Generate	Model Generate	Model Generate
OptNrv_L_PRV	Upper	0	50	120	0	50	120
	Line	Model Generate	Model Generate	Model Generate	Model Generate	Model Generate	Model Generate
OptNrv_R_PRV	Upper	0	50	120	0	50	120
	Line	Model Generate	Model Generate	Model Generate	Model Generate	Model Generate	Model Generate
Retina_L	Upper	0	40	80	0	40	80
	Line	Model Generate	Model Generate	Model Generate	Model Generate	Model Generate	Model Generate
Retina_R	Upper	0	40	80	0	40	80
	Line	Model Generate	Model Generate	Model Generate	Model Generate	Model Generate	Model Generate
Brain	Upper	5	78	50	5	78	50
	Line	Model Generate	Model Generate	Model Generate	Model Generate	Model Generate	Model Generate
Lens_L	Upper	0	6	80	0	6	80
	Line	Model Generate	Model Generate	Model Generate	Model Generate	Model Generate	Model Generate
Lens_R	Upper	0	6	80	0	6	80
	Line	Model Generate	Model Generate	Model Generate	Model Generate	Model Generate	Model Generate

Supplementary Table S4. NRG HN001 Rapid Plan Proton Model Parameters

Structures	Objectives	Volume(%)	Dose(Gy)	Priorities
PTV_5412	Upper	0	60	200
	Lower	99.5	54.12	200
	Lower	97	56	200
	Lower	95	57	200
	Lower	5	59	100
PTV_5940	Upper	0	72	200
	Upper	5	65	100
	Lower	99.5	59	200
	Lower	97	61	200
	Lower	95	63	200
PTV_6270	Upper	0	70	100
	Upper	5	68	100
	Lower	99.5	62.7	200
	Lower	97	64	200
	Lower	95	65	200
PTV_6996	Upper	0	76	250
	Lower	99.5	70	200
	Lower	97	72	200
	Lower	95	74	250
BrachialPlexus	Upper	0	60	80
BrachialPlexus_L	Upper	0	60	80
BrachialPlexus_R	Upper	0	60	80
BrainStem	Upper	0	45	200
	Line	Model Generate	Model Generate	Model Generate
Cochlea_L	Upper	0	50	80
	Mean		40	60
	Line	Model Generate	Model Generate	Model Generate
Cochlea_R	Upper	0	50	80
	Mean		40	60
	Line	Model Generate	Model Generate	Model Generate
Esophagus_UP	Upper	0	45	80
	Line	Model Generate	Model Generate	Model Generate
Eye_L	Upper	0	50	Model Generate
Eye_R	Upper	0	50	Model Generate
LarynxGSL	Mean		35	50
	Line	Model Generate	Model Generate	Model Generate
Lens_L	Upper	0	13	Model Generate
Lens_R	Upper	0	13	Model Generate
Mandible	Upper	0	60	180

	Line	Model Generate	Model Generate	Model Generate
NS	Upper	0	65	250
OpticChiasm	Upper	0	50	100
	Line	Model Generate	Model Generate	Model Generate
OpticNerve_L	Upper	0	50	100
	Line	Model Generate	Model Generate	Model Generate
OpticNerve_R	Upper	0	50	100
	Line	Model Generate	Model Generate	Model Generate
OralCavity	Mean		35	80
	Line	Model Generate	Model Generate	Model Generate
Parotid_L	Mean		23	100
	Line	Model Generate	Model Generate	Model Generate
Parotid_R	Mean		23	100
	Line	Model Generate	Model Generate	Model Generate
SpinalCord	Upper	0	38	200
	Line	Model Generate	Model Generate	Model Generate
TemporalLobe_L	Upper	0	60	100
	Line	Model Generate	Model Generate	Model Generate
TemporalLobe_R	Upper	0	60	100
	Line	Model Generate	Model Generate	Model Generate
TMjoint_L	Upper	0	65	100
	Line	Model Generate	Model Generate	Model Generate
TMjoint_R	Upper	0	65	100
	Line	Model Generate	Model Generate	Model Generate
